# Retinoic acid promotes metabolic maturation of human Embryonic Stem Cell-derived Cardiomyocytes

**DOI:** 10.7150/thno.44146

**Published:** 2020-08-01

**Authors:** Shumei Miao, Dandan Zhao, Xiaoxiao Wang, Xuan Ni, Xing Fang, Miao Yu, Lingqun Ye, Jingsi Yang, Hongchun Wu, Xinglong Han, Lina Qu, Lei Li, Feng Lan, Zhenya Shen, Wei Lei, Zhen-Ao Zhao, Shijun Hu

**Affiliations:** 1 Department of Cardiovascular Surgery of the First Affiliated Hospital & Institute for Cardiovascular Science, Collaborative Innovation Center of Hematology, State Key Laboratory of Radiation Medicine and Protection, Medical College, Soochow University, Suzhou 215000, China.; 2 State Key Laboratory of Stem Cell and Reproductive Biology, Institute of Zoology, Chinese Academy of Sciences, Beijing 100101, China.; 3 University of Chinese Academy of Sciences, Beijing 100049, China.; 4 State Key Laboratory of Space Medicine Fundamentals and Application, China Astronaut Research and Training Center, Beijing 100094, China.; 5 Fuwai Hospital, Chinese Academy of Medical Sciences and Peking Union Medical College, Beijing 100037, China.; 6 Institute of Microcirculation & Department of Pathophysiology of Basic Medical College, Hebei North University, Zhangjiakou 075000, China.

**Keywords:** Embryonic stem cells, Cardiomyocyte differentiation, Retinoic acid, Cardiomyocyte maturation, Oxidative phosphorylation

## Abstract

Cardiomyocytes differentiated from human embryonic stem cells (hESCs) represent a promising cell source for heart repair, disease modeling and drug testing. However, improving the differentiation efficiency and maturation of hESC-derived cardiomyocytes (hESC-CMs) is still a major concern. Retinoic acid (RA) signaling plays multiple roles in heart development. However, the effects of RA on cardiomyocyte differentiation efficiency and maturation are still unknown.

**Methods:** RA was added at different time intervals to identify the best treatment windows for cardiomyocyte differentiation and maturation. The efficiency of cardiomyocyte differentiation was detected by quantitative real-time PCR and flow cytometry. Cardiomyocytes maturation was detected by immunofluorescence staining, metabolic assays and patch clamp to verify structural, metabolic and electrophysiological maturation, respectively. RNA sequencing was used for splicing analysis.

**Results:** We found that RA treatment at the lateral mesoderm stage (days 2-4) significantly improved cardiomyocyte differentiation, as evidenced by the upregulation of *TNNT2*, *NKX2.5* and *MYH6* on day 10 of differentiation. In addition, flow cytometry showed that the proportion of differentiated cardiomyocytes in the RA-treated group was significantly higher than that in control group. RA treatment on days 15-20 increased cardiomyocyte area, sarcomere length, multinucleation and mitochondrial copy number. RNA sequencing revealed RA promoted RNA isoform switch to the maturation-related form. Meanwhile, RA promoted electrophysiological maturation and calcium handling of hESC-CMs. Importantly, RA-treated cardiomyocytes showed decreased glycolysis and enhanced mitochondrial oxidative phosphorylation, with the increased utilization of fatty acid and exogenous pyruvate but not glutamine.

**Conclusion:** Our data indicated that RA treatment at an early time window (days 2-4) promotes the efficiency of cardiomyocyte differentiation and that RA treatment post beating (days 15-20) promotes cardiomyocyte maturation. The biphasic effects of RA provide new insights for improving cardiomyocyte differentiation and quality.

## Introduction

Pluripotent stem cells (PSCs), including embryonic stem cells (ESCs) and induced pluripotent stem cells (iPSCs), hold great potential to differentiate into any cell type of the human body. Human PSC-derived cardiomyocytes (hPSC-CMs) provide the alternative strategies for myocardial repair, drug screening and disease modeling 1, 2. However, the efficiency of cardiomyocyte differentiation is still not stable between cell lines 3, 4, and the immature characteristics of hPSC-CMs impair their applications for drug screening and disease modeling 5, 6.

The mature cardiomyocytes present fully-developed cells that resemble adult cardiomyocytes in structure and function, and have properties including an organized ultrastructure, an increased density of mitochondria, the ability to perform oxidative metabolism using fatty acid, and functional electrophysiology and calcium handling 7-10. However, hPSC-CMs generated by current protocols are more similar to cardiomyocytes at embryonic or early fetal stages 9. Previous reports have shown that electrical stimulation, small molecules, and tissue engineering can promote cardiomyocyte maturation 7, 11-13. However, it is still difficult to obtain fully matured cardiomyocytes from hPSCs, and the mechanism for cardiomyocyte maturation is still largely unknown.

Retinoic acid (RA) is derived from vitamin A through two sequential dehydrogenation reactions, and acts as a ligand of nuclear RA receptors (RARs) 14. Mice with mutant aldehyde dehydrogenase 1 family member A2 (*ALDH1A2*), a rate-limiting enzyme for RA synthesis, showed abnormal heart looping, trabeculation and cardiomyocyte differentiation during heart development 15. In addition, RARs have been proven to be important for heart, outflow tract, and aortic arch development 16. These results suggest that RA plays multiple roles during heart development. Recent studies have mainly focused on the effects of RA on the subtype differentiation of cardiomyocytes. RA treatment with different protocols can promote atrial-like, sinoatrial-like and epicardial-like cell differentiation 1, 17-19. However, the roles of RA in cardiomyocyte maturation remain unclear.

In this study, we found that genes involved in RA synthesis and signaling showed specific expression patterns in different germ layers, providing new clues for understanding mesoderm differentiation. Furthermore, we systemically studied the roles of RA in cardiomyocyte differentiation and maturation. Our results showed that the RA treatment during the time window of the lateral mesoderm stage could promote cardiomyocyte differentiation and that RA treatment post beating could promote maturation of hESC-CMs, as indicated by the increased sarcomere length, mitochondria density, fatty acid metabolism, and the enhanced electrophysiological maturation and calcium handling.

## Materials and Methods

### Mice maintenance and germ layer separation

The ICR mice were housed under specific-pathogen-free (SPF) conditions in the animal facilities at Soochow University. All experimental procedures involving animals were approved by the Laboratory Animal Research Committee of Soochow University. E7.5 embryos were dissected from the deciduas, and the germ layers were separated carefully with glass needles as previously reported 20, 21. Samples were collected for RNA extraction.

### Maintenance and differentiation of hESCs

The hESC lines (NKX2.5^eGFP/w^ HES3, H1 and H9-GCaMP6f) with passages 55-65 were routinely maintained and differentiated as described previously 22, 23. In brief, the hESCs were cultured on feeder-free Matrigel (356231, Corning, USA) in E8 medium (A1517001, Thermo Fisher, USA). Before cardiomyocyte differentiation, the hESCs were passaged every 4 days at 80% confluence using 0.5 mM EDTA and re-plated at a density of 2 × 10^4^ cells/cm^2^. To initiate cardiomyocyte differentiation, the medium was changed with CDM3 (chemically defined medium, three components) containing 5 μM CHIR99021 (C-6556, LC Laboratories, USA) for 48 hours (day 0-2). After a 2-day culture in CDM3, the medium was changed with CDM3 containing 5 μM IWR-1 (I0161, Sigma, USA) for 48 hours (day 4-6). The subsequent culture was in CDM3. The medium was changed every day. Spontaneous beating was commonly observed on day 10. For cardiomyocyte purification, the cells were re-plated on gelatin-coated dishes in RPMI 1640 lacking of glucose and containing 5 mM sodium DL-lactate (L4263, Sigma, USA) from day 11 to day 15 of differentiation. RA (R2625, Sigma, USA) was dissolved in DMSO and added to the CDM3 at the indicated times for cardiomyocyte differentiation and maturation. The final concentration of RA was 1 μM, and the final concentration of DMSO was 0.05% in both groups.

### Reverse transcription and quantitative real-time PCR

TRIzol reagent (15596026, Thermo Fisher, USA) was used to extract total RNA from the different samples. The total RNA was subjected to reverse transcription for single-strand cDNA synthesis using a Takara PrimeScript RT Reagent Kit (RR047A, Takara, Japan). Quantitative real-time PCR (qPCR) assays were performed and the results were analyzed using an Applied Biosystems StepOnePlus Real-Time PCR System (Thermo Fisher, USA). The *18S* rRNA was used as a reference gene for human mRNA quantification, and *Gapdh* was used as a reference gene for mouse mRNA quantification. The data were analyzed using the 2^-ΔΔCT^ method. All primers used for quantitative real-time PCR were listed in Table S1 and Table S2.

### RNA-seq analysis

After purification, the differentiated cardiomyocytes were treated with RA for 5 days and maintained in CDM3 for another 10 days. Cardiomyocytes were then collected in TRIzol reagent for RNA-seq analysis. Briefly, mRNA samples were purified from total RNA using poly-T oligo-attached magnetic beads and subjected to library preparation using NEBNext® UltraTM RNA Library Prep Kit for Illumina^®^ (E7530S, NEB, USA). Then the library preparations were sequenced on an Illumina Hiseq platform in Novogene company (China). The bam files were imported into the IGV software for coverage and splicing visualization. The splicing junctions were normalized using the total mapped reads. The fold change of junction reads/total mapped reads was calculated after RA treatment. Statistical significance was denoted by a fold change > 1.5 or *p* < 0.05, which was calculated with rMATS software.

### Immunofluorescence staining

Cells were fixed in 4% paraformaldehyde for 20 minutes followed by the PBS-T washes. After permeabilization with 0.25% Triton X-100 for 10 minutes, the cells were blocked with 3% BSA for 1 hour at room temperature and incubated with anti-α-actinin antibody (ab9465, Abcam, USA) overnight at 4°C. After rinsing with PBS-T, the cells were incubated with Alexa Fluor 594 secondary antibody (715-585-151, Jackson ImmunoResearch, USA) at room temperature for 1 hour, and then the nuclei were stained with Hoechst 33342 (14533, Sigma, USA). Fluorescence images were acquired with an LSM 880 confocal laser scanning microscope (Zeiss, Germany). Each cardiomyocyte was analyzed using ImageJ software with standard analysis plugins for average sarcomere length and cell area. More than 60 cells were analyzed for each group 6, 13. The antibodies used in this study are listed in Table S3.

### Flow cytometry

Differentiated cells were dissociated into single cells with 0.25% trypsin-EDTA and fixed in 1% paraformaldehyde for 10 minutes. The anti-cardiac troponin T (TNNT2) antibody (MS-295-P1, Thermo Fisher, USA) and Alexa Fluor 647 secondary antibody (715-605-151, Jackson ImmunoResearch, USA) were used for flow cytometry analysis. Data were acquired and analyzed using Guava easyCyte^TM^ 8 (EMD Millipore, Germany).

### Mitochondrial staining

hESC-CMs were plated on the coverslips coated with 0.1% gelatin and cultured for 72 hours before staining. Mitochondrial staining was performed by incubating cells with prewarmed medium supplemented with 0.1 μM MitoTracker Red CMXRos for 20 minutes. After 3 washes with D-PBS, the cells were fixed and subsequently incubated with TNNT2 antibody, Alexa Fluor 488 secondary antibody (715-545-151, Jackson ImmunoResearch, USA) and Hoechst 33342 as described above. Mitochondria were visualized using an LSM 880 confocal laser scanning microscope (Zeiss, Germany).

### MitoTracker Red flow cytometry

Cardiomyocytes were washed with D-PBS and then incubated with prewarmed medium supplemented with 0.1 μM MitoTracker Red CMXRos (M7512, ThermoFisher Scientific, USA) for 20 minutes. After 3 washes with D-PBS, the cells were dissociated into single cells using 0.25% trypsin-EDTA and fixed with 1% paraformaldehyde for 20 minutes. Then, the cells were washed with D-PBS and analyzed with Millipore Guava easyCyte 8 (EMD Millipore, USA).

### Metabolic assays

A Seahorse XF24 Extracellular Flux Analyzer (Seahorse Bioscience, USA) was used to assess oxygen consumption rates (OCR) and extracellular acidification rates (ECAR). On day 30 of differentiation, cardiomyocytes were seeded onto the plates coated with 0.1% gelatin at 5 × 10^5^ cells/well. The Seahorse assays were carried out after 3 days of culture in an XF24 24-well plate. For the mitochondrial metabolism assay, one hour before the assay, the culture medium was re-placed with pH 7.4 unbuffered assay medium supplemented with 5.5 mM glucose (G7021, Sigma, USA), 1 mM sodium pyruvate (11360070, Thermo Fisher, USA), 2 mM GlutaMAX (35050061, Thermo Fisher, USA) or a combination of these substances. For the palmitate metabolism assay, cardiomyocytes were cultured in substrate-limited medium (DMEM, 0.5 mM glucose, 1 mM GlutaMAX, 0.5 mM carnitine (C0283, Sigma, USA), 1%FBS) for 24 hours. After 2 washes with FAO assay medium (111 mM NaCl, 4.7 mM KCl, 1.25 mM CaCl_2_, 2 mM MgSO_4_, 1.2 mM NaH_2_PO_4_, 0.5 mM carnitine and 5 mM HEPES; adjusted to PH 7.4 with NaOH), the cells were incubated in FAO assay medium for 45 minutes prior to the assay in a non-CO_2_ incubator. Then, 40 μM Etomoxir (ETO, an inhibitor of carnitine palmitoyltransferase-1) (E1905, Sigma, USA) or vehicle was added to each well for 15 minutes before starting the assay. The palmitate assay was started just after the addition of 200 μM sodium palmitate (P9767, Sigma, USA) in 0.1% BSA. Seahorse XF Cell Mito Stress Test compounds (103015-100, Agilent, USA) were added sequentially to achieve final concentrations of 2 μM oligomycin, 1.5 μM FCCP, and 2 μM rotenone/antimycin A (Rtn/AA). ETO-responsive OCR changes were used to confirm fatty acid metabolism. For the glycolysis assay, the culture media was re-placed with unbuffered assay medium. Glycolysis stress test compounds (103020-100, Agilent, USA) were added to achieve final concentrations of 10 mM glucose, 2 μM oligomycin, and 5 mM 2-DG. The OCR and ECAR values were further normalized to 10 μg of protein in each well. The protein concentrations were measured with a BCA kit (P0011, Beyotime Biotechnology, China).

### Electrophysiology

Action potentials (APs) recorded from spontaneous cardiomyocytes were conducted using the Axopatch 200B Microelectrode Amplifier (Molecular Devices, USA) in the current clamp mode. For whole-cell recording, cardiomyocytes were incubated in the bath solution containing 140 mM NaCl, 4 mM KCl, 1.2 mM CaCl_2_, 1 mM MgCl_2_, 10 mM HEPES, and 10 mM glucose, with pH adjusted to 7.4 with NaOH. The pipette solution consisted of 115 mM potassium aspartate, 15 mM KCl, 4 mM NaCl, 1 mM MgCl_2_, 5 mM Mg-ATP, 5 mM HEPES, and 5 mM EGTA, with pH adjusted to 7.2 with KOH. Data acquisition was performed using pClamp software. The action potential duration at 50% (APD_50_) and 90% (APD_90_) repolarization were calculated by Clampfit 10.3 software.

### Calcium (Ca^2+^) handling assay

H9-GCaMP6f hESC line was generated as previously report 24, and the differentiated cardiomyocytes were seeded onto confocal dishes. Intracellular calcium flux was captured using an LSM 880 confocal laser scanning microscope (Zeiss, Germany). Spontaneous Ca^2+^ transients were acquired by line scan mode with a sampling rate of 1 ms/line under the condition of 37 °C and 5% CO_2_. The amplitude of Ca^2+^ transient was expressed as normalized fluorescence signals (∆F/F0). The time to peak and decay time were recorded. All of these measurements were acquired for at least four beats in each video and averaged for comparison.

### Chromatin immunoprecipitation (ChIP)-PCR

The ChIP assay was performed using EZ-ChIP kit (17-371, Millipore, USA) according to the manufacturer's protocol. Briefly, ~10^7^ cardiomyocytes were fixed with 1% formaldehyde to covalently crosslink proteins to DNA, followed by addition of glycine (0.125 M) to quench unreacted formaldehyde. After washed with cold PBS, cells were lysed in SDS lysis buffer and sonicated (10-12 sets of 15 seconds each at 15-second interval) on ice. Sheard crosslinked chromatin were immunoprecipitated with anti-RARα antibody (ab41934, Abcam, USA). Antibodies against RNA Polymerase II and mouse IgG in the EZ-ChIP kit were added as positive and negative control respectively. After DNA purification, semi-quantitative PCR was performed with primers specific to the two retinoic acid response elements (RAREs) in the promoter region of human *PGC1A*. The two RAREs were named as RARE1 (-229 bp ~ -212 bp: 5'-AGGGTTATCTGGGGGCGA-3') and RARE2 (-70 bp ~ -53 bp: 5'-TGACTCTGAGATGCCCTC-3'), respectively. The matched sequences of RAREs in *PGC1A* promoter were acquired from the JASPAR database. The primers used for ChIP-PCR were listed in Table S4, while primers for human *GAPDH* in EZ-ChIP kit were used as control primers.

### Statistical analysis

Comparisons between two groups were performed using Student's *t*-test. Comparisons among multiple groups were performed with one-way analysis of variance (ANOVA). Statistical significance was denoted by a *p* < 0.05. All data were presented as the mean ± SEM. All experiments were performed at least three times.

## Results

### Specific gene expression patterns of the RA pathway in E7.5 mouse embryos

**Figure 1:**
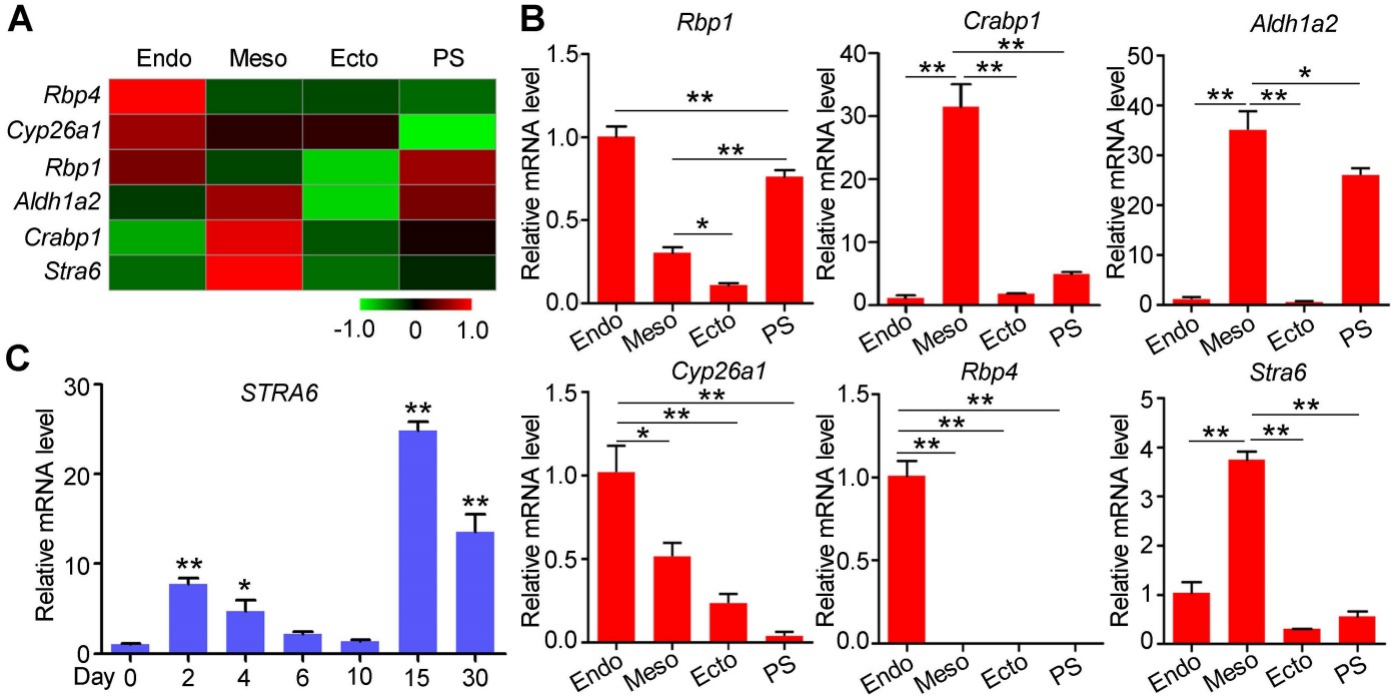
** Specific gene expression patterns of the RA pathway in E7.5 mouse embryos and during human cardiomyocyte differentiation.** (**A**) Heatmap showed the expression patterns of genes involved in RA synthesis and degradation in the endoderm (Endo), mesoderm (Meso), ectoderm (Ecto) and primitive streak (PS) from E7.5 mouse embryos. (**B**) Real-time PCR showed the expression of RA synthesis and degradation genes in mouse Endo, Meso, Ecto and PS on E7.5. (**C**) Real-time PCR showed a biphasic expression pattern of *STRA6* during cardiomyocyte differentiation from hESCs. One-way ANOVA; *p<0.05; **p<0.01.

Retinol can be carried by retinol-binding protein 4 (*Rbp4*), and transferred into cytoplasm by a receptor named as stimulated by retinoic acid 6 (*Stra6*) 25. After a series of dehydrogenation reactions, retinol is metabolized into all-trans retinoic acid (RA). RA regulates the transcriptional activity of target genes as a ligand for RAR/RXR heterodimers. In our previous study, we performed transcriptome analysis on E7.5 germ layers to identify functional genes for embryonic development and stem cell differentiation 20, 26. The specific expressions of the germ layer markers indicated the embryo tissues were separated successfully (Figure S1). Among the differentially expressed genes, RA pathway genes showed specific expression patterns in three germ layers (Figure 1A-B). *Rbp4* and *Stra6* were highly expressed in the endoderm (Endo) and mesoderm (Meso), respectively, suggesting that RA synthesis depends on the cooperation of the endoderm and mesoderm. Furthermore, compared with the ectoderm (Ecto), the primitive streak (PS) highly expressed *Rbp1*, *Aldh1a2*, and *Crabp1*, which are involved in RA generation and signal transduction. These genes continued to be expressed in the mesoderm (Figure 1A-B). Thus, the RA pathway may have been primed in the primitive streak by the expression of RA synthases, and be triggered by *Stra6*, which was highly expressed in the mesoderm compared to primitive streak. In addition, *Cyp26a1*, a gene for RA degeneration, was mainly expressed in the endoderm (Figure 1A-B). These results suggest that RA pathway may play important roles in mesoderm differentiation. Since specific *STRA6* expression may provide clues for optimal time of RA treatment, we performed qPCR to detect its mRNA levels during cardiomyocyte differentiation of hESCs. *STRA6* mRNA was elevated at the mesoderm stage (days 2-4) and was increased dramatically in beating cardiomyocytes on day 15 of cardiomyocyte differentiation compared to undifferentiated cells (Figure 1C). Collectively, these results reveal that RA might play biphasic roles at these two specific stages during the differentiation of hESCs into cardiomyocytes.

### RA treatment on days 2-4 is important for cardiomyocyte differentiation

With regards to the gene expression of RA pathway members, we focused on the effects of RA on the mesoderm and downstream cardiomyocyte differentiation. Firstly, RA was supplemented at different stages of cardiomyocyte differentiation (Figure S2A). The addition of RA during the first two days (days 0-2) dramatically inhibited the differentiation of the entire primitive streak (PS). Markers for the anterior PS (*FOXA2* and *GSC*), shared PS (*T* and *MIXL1*) and mid/posterior PS (*MESP1* and *EVX1*) were significantly decreased upon RA treatment (Figure S2B). RA treatment from day 2 to day 4 significantly inhibited the paraxial mesoderm marker *DLL1*, but enhanced the expression of lateral mesoderm markers (*FOXF1*, *KDR* and *PDGFRA*) (Figure S2C). RA treatment from day 4 to day 6 substantially inhibited the expression of cardiac progenitor markers (*ISL1*, *NKX2.5* and *TBX20*) and limb bud markers (*PRRX1* and *HOXB5*) (Figure S2D), consistent with previous publications 27-29. RA treatment from day 6 to day 10 significantly inhibited endothelial markers (*CD31*, *CD34* and *CD144*) and myocardial markers (*NKX2.5*, *TNNT2*, *MYH7* and *MYH6*), but the epicardial marker *TBX18* was increased by RA treatment (Figure S2E), consistent with a previous study 18. Taken together, the results suggest that RA mainly plays negative roles in cardiomyocyte differentiation but promotes lateral mesoderm differentiation, consistent with the *STRA6* expression at the mesoderm stage (days 2-4) of hESC differentiation and in E7.5 mouse mesoderm *in vivo*. Therefore, we assumed that RA treatment on days 2-4 should promote cardiomyocyte differentiation.

**Figure 2:**
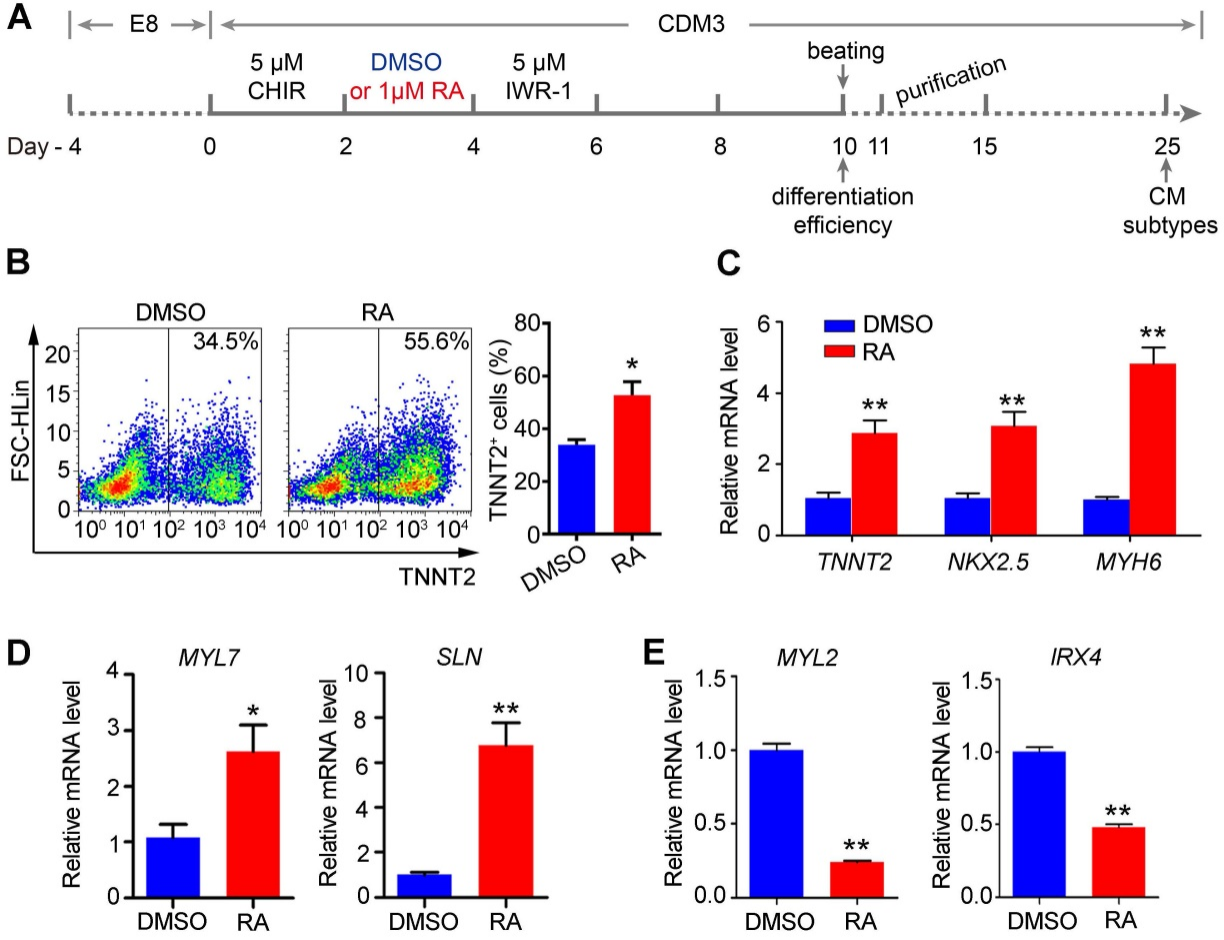
** RA treatment on days 2-4 promotes cardiomyocyte differentiation.** (**A**) Schematic diagram of cardiomyocyte differentiation from hESCs. (**B**) Flow cytometry analysis of differentiated cardiomyocyte (day 10) showed that the proportion of TNNT2^+^ cells was increased after RA treatment. (**C**) Real-time PCR showed that RA promoted the expression of cardiomyocyte-specific markers on day 10 of differentiation. (**D-E**) Real-time PCR showed the expression patterns of the atrium-specific markers (*MYL7* and *SLN*) and ventricular markers (*MYL2* and *IRX4*) on day 25 of differentiation. Student's *t*-test; *p<0.05; **p<0.01.

Flow cytometry analysis confirmed that RA treatment at the mesoderm stage (days 2-4) promoted cardiomyocyte differentiation, as evidenced by the increased proportion of TNNT2^+^ cells on day 10 of differentiation in H1 hESC line (Figure 2A-B). Furthermore, qPCR confirmed the upregulation of cardiomyocyte markers *TNNT2*, *NKX2.5* and *MYH6* (Figure 2C). Additionally, we used another hESC line, NKX2.5^eGFP/w^ hES3 to examine cardiomyocyte differentiation supplemented with RA on days 2-4 30. Similarly, RA treatment at this stage could promote gene expression of the lateral mesoderm markers on day 4 (Figure S3A) and increased cardiomyocyte proportions (eGFP^+^) on day 10 of differentiation (Figure S3B). Collectively, the results suggest that RA treatment on days 2-4 promotes lateral mesoderm differentiation, and subsequently enhances cardiomyocyte differentiation from hESCs.

In addition, we noticed that RA treatment (days 2-4) upregulated atrium-specific markers (*MYL7* and *SLN*) on day 25 of differentiation, while ventricular markers (*MYL2* and *IRX4*) were downregulated by RA treatment (Figure 2D-E). These results support that RA signaling at the lateral mesoderm stage of development is important for atrial differentiation. Therefore, RA treatment at the lateral mesoderm stage not only promotes the efficiency of cardiomyocyte differentiation but also atrial specification.

### RA treatment on days 15-20 promotes RNA isoform switch and RNA splicing

**Figure 3:**
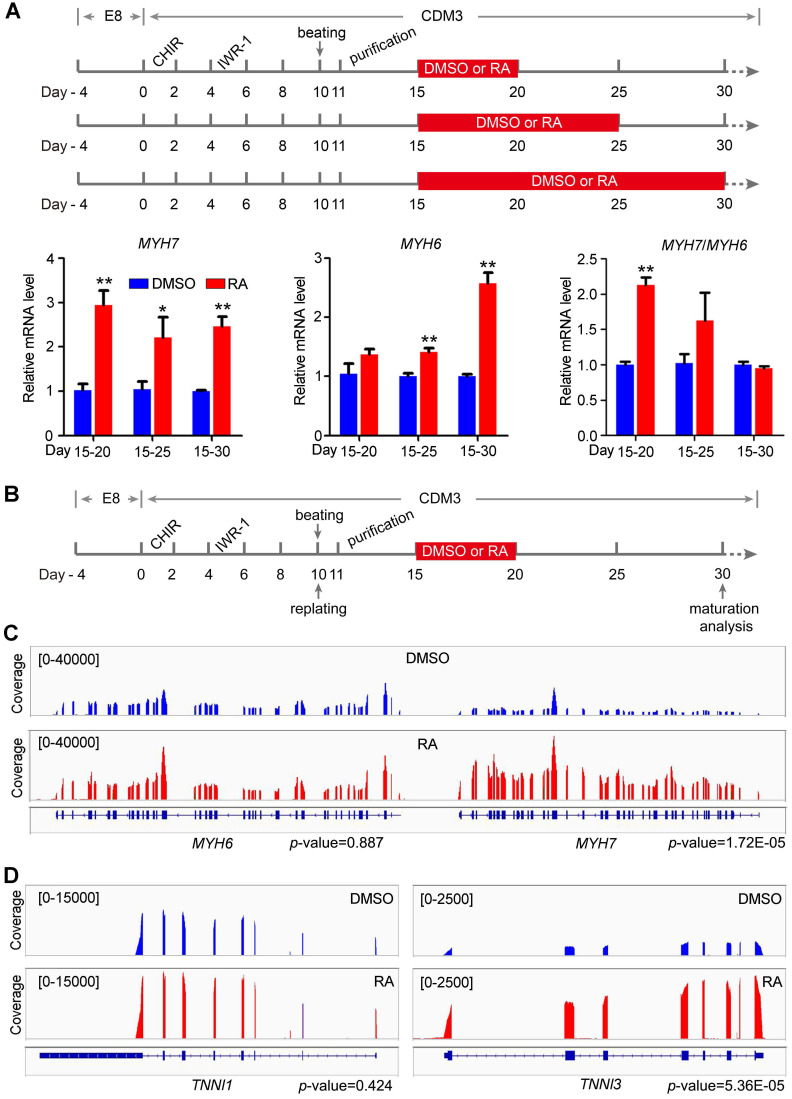
** RA promotes RNA isoform switch to the maturation-related form.** (**A**) Schematic diagram of cardiomyocyte maturation induction. Purified cardiomyocytes were treated with RA at different time intervals. All samples were harvested on day 30. Real-time PCR showed the expression patterns and ratio of the maturation-related genes *MYH7* and *MYH6* after RA treatment at different time intervals. (**B**) Defined schematic diagram for purification and maturation of cardiomyocytes with RA treatment. **(C)** RNA-seq coverage showed the increased *MYH7*/*MYH6* ratio in RA-treated hESC-CMs. **(D)** RNA-seq coverage showed the increased *TNNI3*/*TNNI1* ratio in RA-treated hESC-CMs.

The increased *STRA6* mRNA expression post beating indicated that the RA pathway might play an important role in the maturation of hESC-CMs. For maturation analysis, hESC-CMs were purified in CDM3 containing lactic acid and lacking of D-glucose for 4 days to remove noncardiomyocytes. Thereafter, we optimized the time window of RA treatment for cardiomyocyte maturation as indicated in Figure 3A. With the development of human heart and the maturation of human cardiomyocytes, the ratio of *MYH7*/*MYH6* increases and *MHY7* becomes the dominant isoform in human adult heart 5, 7, 10. Thus, ratio of *MYH7*/*MYH6* was widely used as a reliable indicator for cardiomyocyte maturation 7, 31, 32. Quantitative real-time PCR showed that *MYH7*/*MYH6* ratio was significantly increased in cardiomyocytes treated with RA on days 15-20 (Figure 3A). Thus, RA treatment on days 15-20 might accelerate cardiomyocyte maturation following the protocol in Figure 3B. Meanwhile, we analyzed the isoform switch using RNA-seq data. Genome browser snapshots confirmed the increased ratio *MYH7/MYH6* by RA treatment (Figure 3C). Furthermore, *TNNI3/TNNI1* ratio, another indicator of cardiomyocyte maturation 32, was also increased (Figure 3D). Moreover, RA treatment upregulated the expression of maturation marker genes related to calcium handling (*RYR2*, *SERCA2*, *CACNA1C*) and electrical conduction (*KCNJ2*, *SCN5A*) in hESC-CMs (Figure S4), consistent with cardiomyocyte maturation phenotypes in previous reports 7, 33. These results were further verified in cardiomyocytes derived from H1 hESC line (Figure S5).

**Figure 4:**
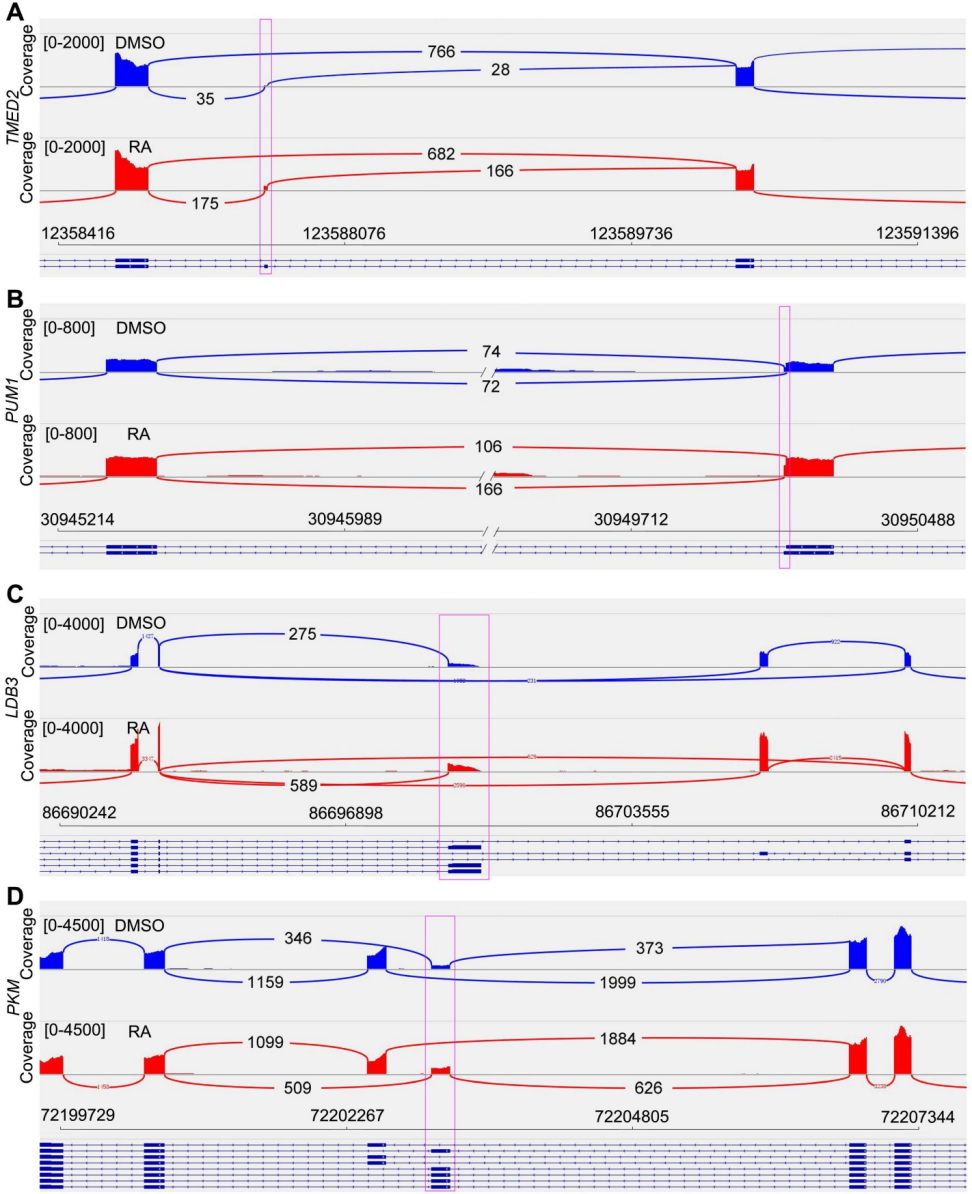
** Sashimi Plot shows the alternative splicing of maturation-related genes.** (**A**) Sashimi Plot showed the reduced exon skipping of* TMED2* gene in RA-treated cardiomyocytes. (**B**) Sashimi Plot showed alternative 3' splice site (A3SS) of* PUM1* gene. (**C**) RA promoted the expression of short *LDB3*. (**D**) Sashimi Plot showed alternative splicing of *PKM* gene, which had mutually exclusive exon 9 and 10.

RNA splicing is a crucial layer in gene expression, which is important for cardiomyocyte maturation 34. Previous studies have found some maturation-related splicing genes, including *TMED2*, *PUM1*, *LDB3* and *PKM* 34-38. Our RNA-seq data provided the possibility for RNA splicing analysis. After RA treatment, we found *TMED2* showed reduced exon skipping (Figure 4A, Figure S6A). *PUM1* had alternative 3' splice site (A3SS), and RA treatment promoted the selection of a longer *PUM1* variant (Figure 4B, Figure S6B). The short form of *LDB3* was increased in adult heart. In our study, RA-induced short *LDB3* up-regulation was consistent with cardiomyocyte maturation (Figure 4C, Figure S6C). The *PKM* gene has mutually exclusive exons. We observed increased splicing of *PKM1* after RA treatment (Figure 4D, Figure S6D), which is the adult variant of pyruvate kinase. Therefore, RA promotes cardiomyocyte maturation at RNA splicing level.

### RA treatment on days 15-20 promotes structural maturation of hESC-CMs

**Figure 5:**
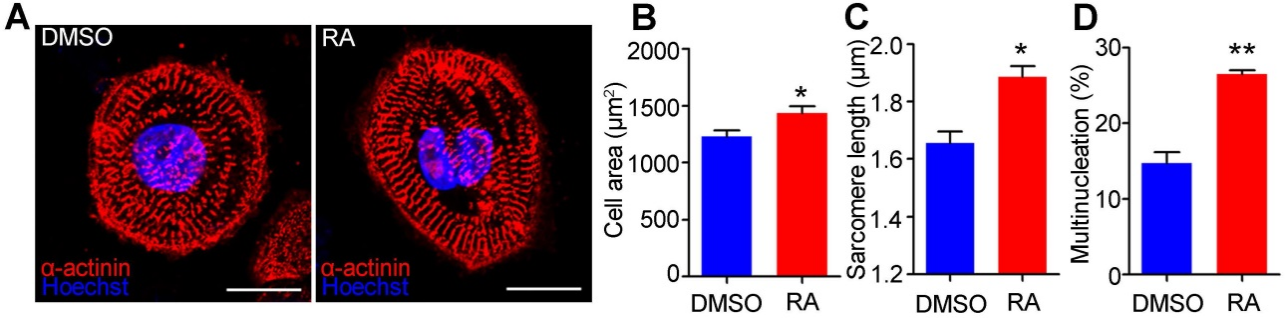
** RA promotes structural maturation of hESC-CMs.** (**A**) Immunostaining of sarcomeric α-actinin (red) and nuclear counterstaining with Hoechst 33342 (Blue) in DMSO- and RA-treated hESC-CMs. Scale bars, 50 µm. (**B**) Quantitative analyses of immunostaining showed that cell area was increased in RA-treated hESC-CMs. (**C**) Quantitative analyses of immunostaining showed that sarcomere length was increased in RA-treated hESC-CMs. (**D**) Quantitative analyses of immunostaining showed that multinucleation was increased in RA-treated hESC-CMs. n > 50 for each group. Student's *t*-test; *p<0.05; **p<0.01.

The morphology and structure of hESC-CMs on day 30 were further analyzed to confirm the mature phenotypes. Immunostaining with a sarcomeric α-actinin antibody indicated that RA treatment could substantially increase sarcomere length (Figure 5A). Quantitative analyses showed that RA-treated hESC-CMs exhibited significant increases in cell area (1436 ± 57 μm^2^ in the RA group versus 1230 ± 51μm^2^ in the DMSO group, *p*<0.05) (Figure 5B) and sarcomere length (measured by the distance between z-disks, 1.89 ± 0.04 μm in the RA group versus 1.66 ± 0.04 μm in the DMSO group, *p*<0.05) (Figure 5C). The percentage of multinucleated cardiomyocytes was significantly increased by RA treatment (Figure 5D). Taken together, these data demonstrate that RA treatment promotes the structural maturation of cardiomyocytes.

### RA promotes electrophysiological maturation and calcium handling of hESC-CMs

**Figure 6:**
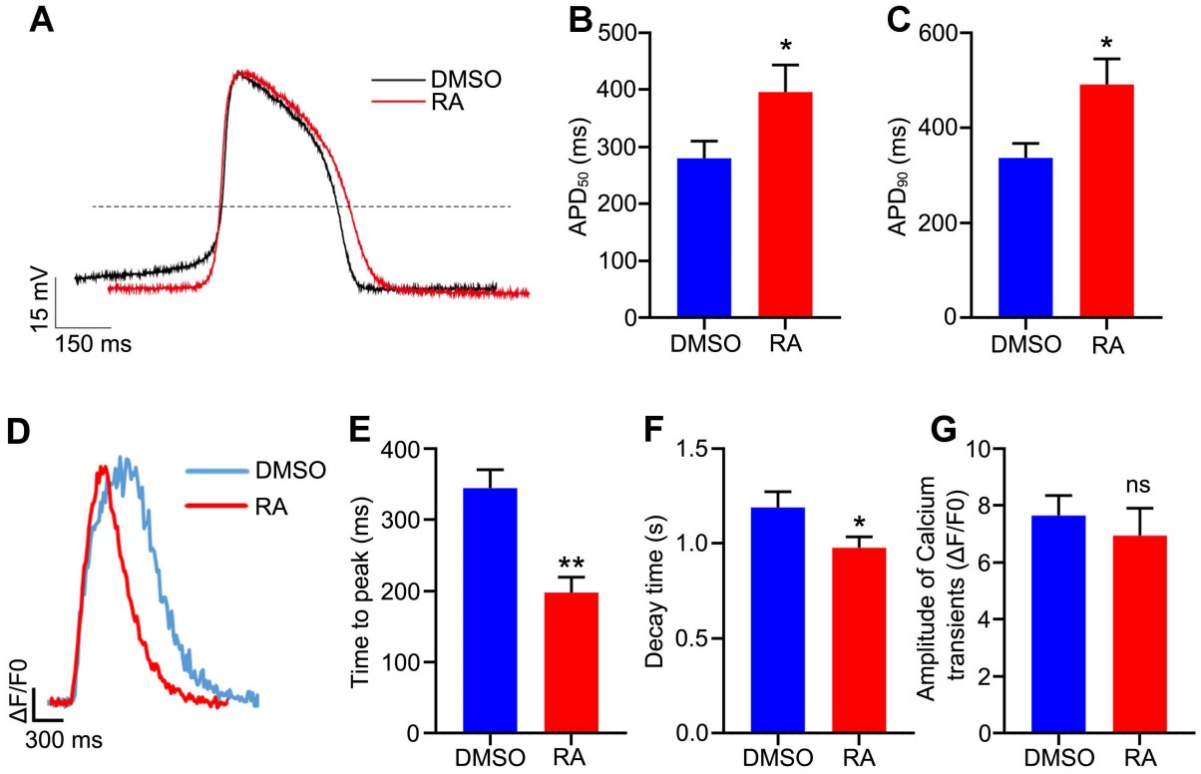
**RA promotes electrophysiological maturation and calcium handling of hESC-CMs.** (**A**) Representative spontaneous action potential recordings from the DMSO and RA-treated hESC-CMs. (**B-C**) APD_50_ (B) and APD_90_ (C) were increased in RA-treated hESC-CMs. n=21 for DMSO group and n =22 for RA group. (**D**) Representative intracellular calcium transients from DMSO and RA-treated hESC-CMs. (**E-F**) The time to peak (E) and decay time (F) were decreased in RA-treated hESC-CMs. n=19 for each group. (**G**) The amplitude of calcium transient was comparable in the two groups. Student's *t*-test; *p<0.05; **p<0.01, and ns, not significant.

To further verify the functional maturity of the hESC-CMs at an electrophysiological level, action potential was measured with patch clamp technique. The action potential duration at 50% repolarization (APD_50_) and APD_90_ were usually used to verify the maturity of cardiomyocytes 6, 39, 40. In our study, APD_50_ was significantly prolonged in the RA-treated hESC-CMs (395.8 ± 47.7 ms in the RA group versus 280.4 ± 30.2 ms in the DMSO group, *p*<0.05), and APD_90_ showed the similar trend after RA treatment (491.1 ± 54.3 ms in the RA group versus 337.0 ± 30.6 ms in the DMSO group, *p*<0.05) (Figure 6A-C). These results were consistent with previous reports 6, 39, 40, implying RA promoted cardiomyocyte electrophysiological maturity.

To further examine the calcium handling of the hESC-CMs, we performed cardiomyocyte differentiation and calcium imaging using H9-GCaMP6f, which was the H9 hESC line with green fluorescent calcium-modulated protein 6 fast type (GCaMP6f) calcium sensor in the AAVS1 locus 24. On day 30 of differentiation, calcium transients for the DMSO and RA groups were recorded. In the RA-treated CMs, the time to peak was decreased significantly (197.8 ± 21.7 ms in the RA group versus 344.6 ± 25.8 ms in the DMSO group, *p*<0.01; Figure 6D-E). Meanwhile, the decay time were also decreased significantly after RA treatment (0.98 ± 0.06 sec in the RA group versus 1.19 ± 0.08 sec in the DMSO group, *p*<0.05; Figure 6F). The amplitude of Ca^2+^ transient was unchanged by RA treatment (Figure 6G). Consistent with previous reports 9, 10, 40, these results indicate RA treatment promotes hESC-CM functional maturation, in term of electrophysiology and calcium handling.

### RA promotes the metabolic switch from glycolysis to oxidative phosphorylation

**Figure 7:**
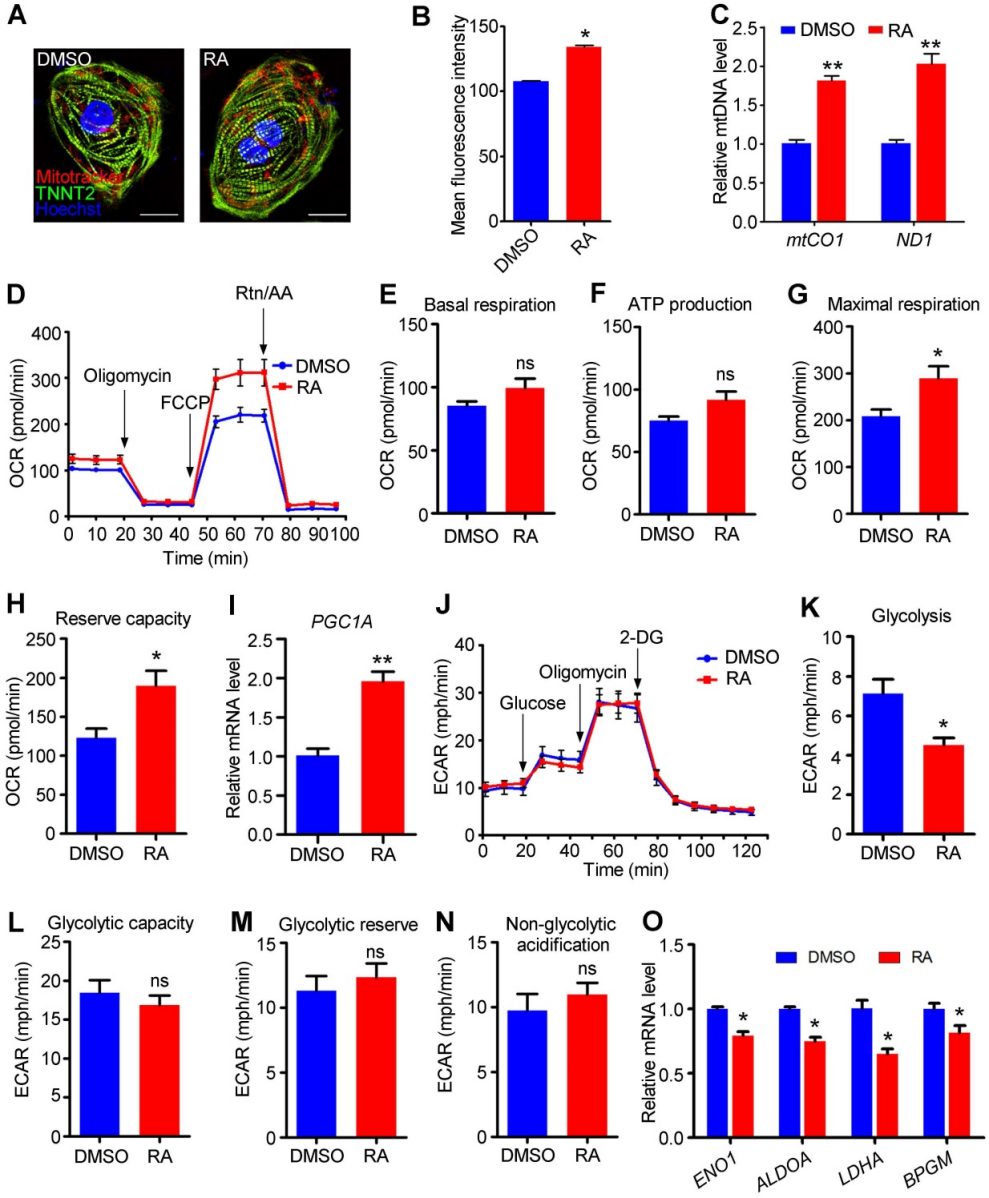
**RA promotes the metabolic switch from glycolysis to oxidative phosphorylation.** (**A**) The distribution of mitochondria was not altered in RA-treated hESC-CMs. MitoTracker Red CMXRos (red), TNNT2 (green) and Hoechst 33342 nuclear stain (blue) were shown. (**B**) Flow cytometry showed that RA promoted the mitochondrial content of hESC-CMs. (**C**) Real-time PCR showed that the mitochondrial DNA copy number, calculated as mtCO1 or ND1/nuclear DNA content, was significantly higher in RA-treated hESC-CMs than in DMSO-treated hESC-CMs. **(D)** Representative OCR traces of the DMSO- and RA-treated hESC-CMs in the presence of both glucose and pyruvate obtained using a Seahorse XF24 Extracellular Flux Analyzer. **(E-H)** Quantification of basal respiration (E), ATP production (F), maximal respiration (G) and reserve capacity (H) in the DMSO- and RA-treated hESC-CMs in the presence of both glucose and pyruvate. **(I)** Real-time PCR showed increased *PGC1A* mRNA expression in RA-treated hESC-CMs. (**J**) Representative ECAR traces of DMSO- and RA-treated hESC-CMs obtained to investigate glycolysis using a Seahorse XF24 Extracellular Flux Analyzer. (**K-N**) Quantification of the glycolysis (K), glycolytic capacity (L), glycolytic reserve (M) and non-glycolytic acidification (N) in DMSO- and RA-treated hESC-CMs. (**O**) Real-time PCR showed that glycolysis-related genes were significantly downregulated after RA treatment. All measurements were normalized to the 10 µg of protein. Student's *t*-test; *p<0.05; **p<0.01, and; ns, not significant.

Adult cardiomyocytes preferentially use oxidative phosphorylation for energy generation 12. Immunostaining using MitoTracker Red CMXRos showed that the distribution of mitochondria was not affected by RA treatment (Figure 7A), but the mitochondrial mass of hESC-CMs was significantly increased in RA-treated hESC-CMs compared to DMSO-treated hESC-CMs, as detected by flow cytometry (Figure 7B). The levels of* mtCO1* and *ND1* DNA are commonly used to evaluate mitochondrial DNA (mtDNA) 32, and our data indicated that mtDNA levels were substantially increased in RA-treated hESC-CMs compare to DMSO-treated hESC-CMs (Figure 7C).

Subsequently, we sought to determine whether the increased mitochondrial mass could enhance mitochondrial metabolism in RA-treated hESC-CMs. To analyze the mitochondrial metabolism, a Seahorse metabolic flux assay was performed with sequential additions of a mitochondrial ATP synthase inhibitor (oligomycin), a proton gradient discharger (FCCP), and electron inhibitors (rotenone/antimycin A), with D-glucose and sodium pyruvate in the detection medium (Figure 7D). Both the basal respiration and ATP production were unaffected, but the mitochondrial maximal respiration capacity and reserve capacity were significantly higher, as measured by the oxygen consumption rate (OCR), in RA-treated hESC-CMs than control (Figure 7E-H). Additionally, in H1 derived-cardiomyocytes, RA significantly promoted basal respiration, ATP production, mitochondrial maximal respiration capacity and reserve capacity (Figure S7). *PGC1A* is a mitochondrial activity-related gene 41. We found *PGC1A* was significantly increased in RA-treated hESC-CMs (Figure 7I). ChIP-PCR demonstrated the direct binding of RARα to RAREs in *PGC1A* promoter after RA treatment (Figure S8). Collectively, these results demonstrate that RA promotes oxidative phosphorylation in hESC-CMs. We next investigated glycolysis in hESC-CMs using a Seahorse XF assay to assess extracellular acidification rates (ECAR). RA treatment substantially decreased glycolysis in hESC-CMs (Figure 7J-K), while the glycolytic capacity, glycolytic reserve and non-glycolytic acidification were not affected (Figure 7L-N). The downregulation of genes involved in glycolytic metabolism, such as* ENO1*, *ALDOA*, *LDHA* and *BPGM*, supported the decreased glycolysis in RA-treated hESC-CMs (Figure 7O). Together, these data demonstrate that RA promotes a metabolic switch from glycolysis to mitochondrial metabolism.

### RA promotes fatty acid and pyruvate metabolism in the mitochondria of hESC-CMs

**Figure 8:**
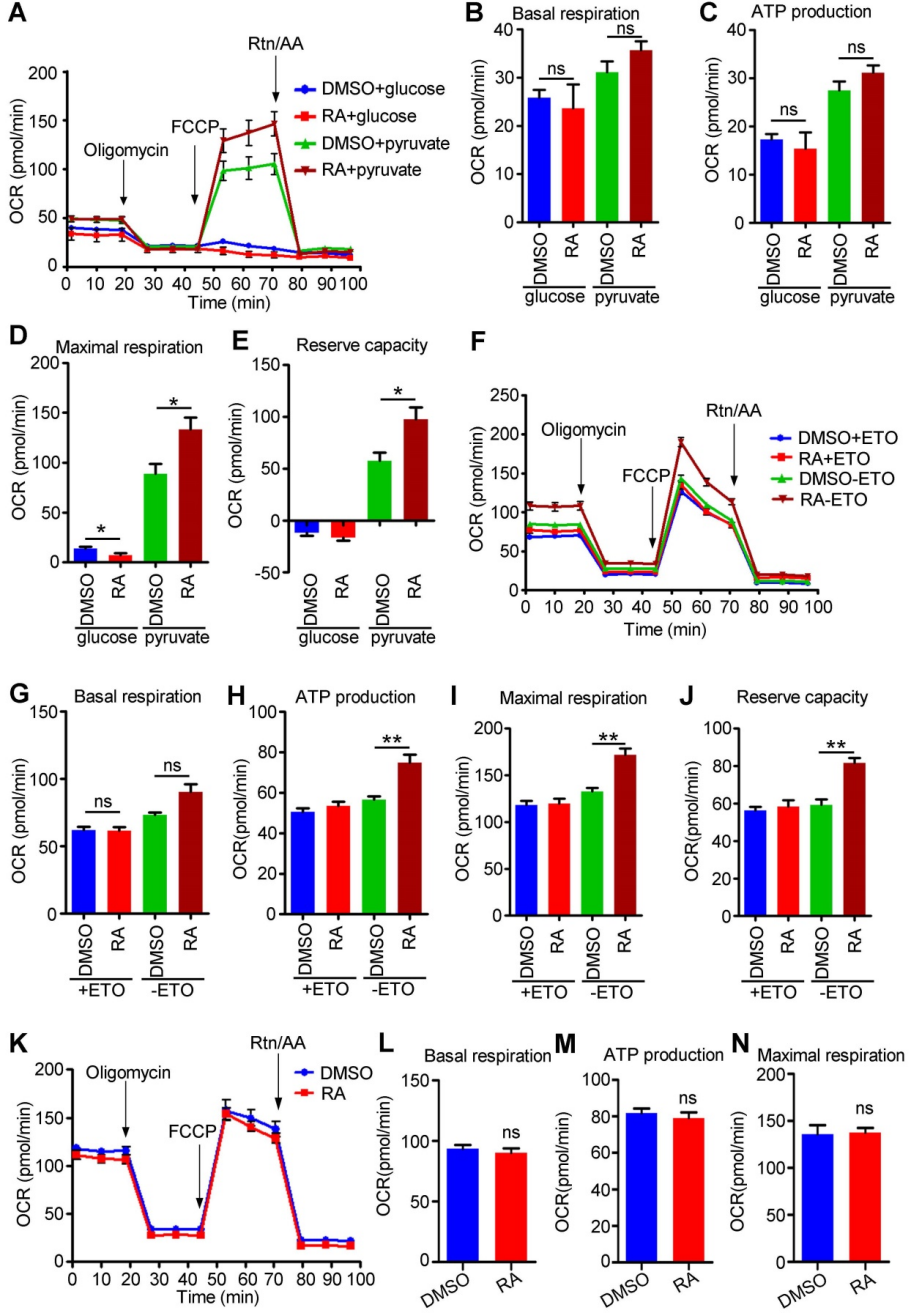
** RA promotes mitochondrial metabolism via fatty acids and pyruvate.** (**A**) Representative OCR traces of DMSO- and RA-treated hESC-CMs in the presence of either glucose or pyruvate. **(B-E)** Quantification of basal respiration (B), ATP production (C), maximal respiration (D) and reserve capacity (E) in DMSO and RA-treated hESC-CMs in the presence of either glucose or pyruvate. (**F**) Representative OCR traces of DMSO- or RA-treated hESC-CMs in the presence of sodium palmitate obtained to assess fatty acid metabolism. ETO-responsive OCR changes were used to confirm fatty acid metabolism. **(G-J)** Quantification of basal respiration (G), ATP production (H), maximal respiration (I) and reserve capacity (J) in DMSO- and RA-treated hESC-CMs in the presence of sodium palmitate to assess fatty acid metabolism. (**K**) Representative OCR traces of DMSO- or RA-treated hESC-CMs in the presence of GlutaMAX obtained using a Seahorse XF24 Extracellular Flux Analyzer. **(L-N)** Quantification of the basal respiration (L), ATP production (M) and maximal respiration (N) in DMSO- and RA-treated hESC-CMs in the presence of GlutaMAX. All measurements were normalized to the 10 µg of protein. Student's *t*-test; *p<0.05; **p<0.01, and ns, not significant.

The energy sources of mitochondria mainly include pyruvate derived from glucose, acyl-CoA derived from fatty acids, and amino acids (such as glutamine). Although glycolysis was decreased in RA-treated hESC-CMs compared to DMSO-treated hESC-CMs, mitochondrial metabolism was significantly increased when the medium was supplemented with both glucose and pyruvate (Figure 7D), indicating that pyruvate might be the main energy source for mitochondria. We further detected OCR by supplying cells with either glucose or pyruvate. Interestingly, hESC-CMs preferred pyruvate over glucose. Furthermore, we found that glucose usage was decreased but that pyruvate usage was significantly increased in RA-treated hESC-CMs compared to DMSO-treated hESC-CMs (Figure 8A-E). Thus, exogenous pyruvate is an efficient substrate for acetyl-CoA production and the subsequent TCA cycle.

Mature cardiomyocytes mainly use fatty acids as energy resources for oxidative phosphorylation 42. To examine the fatty acid metabolism in RA-treated hESC-CMs, we performed an FAO assay in the presence of palmitate. We found that the oxidation of palmitate was significantly higher in RA-treated hESC-CMs than in DMSO-treated hESC-CMs, as evidenced by the increased ATP production, maximal respiration and reserve capacity after RA treatment (Figure 8F-J). As an inhibitor of carnitine palmitoyltransferase-1, ETO inhibited fatty acid metabolism. RA-treated hESC-CMs showed increased ETO-responsive OCR change (Figure 8H-J), indicating RA could promote fatty acid metabolism.

Glutamine, which is widely used in cell culture, as an amino acid energy resource, can be catabolized to α-ketoglutarate and support TCA cycle anaplerosis. We found that the utilization of glutamine was comparable between RA-treated hESC-CMs and DMSO-treated hESC-CMs (Figure 8K-N).

**Figure 9:**
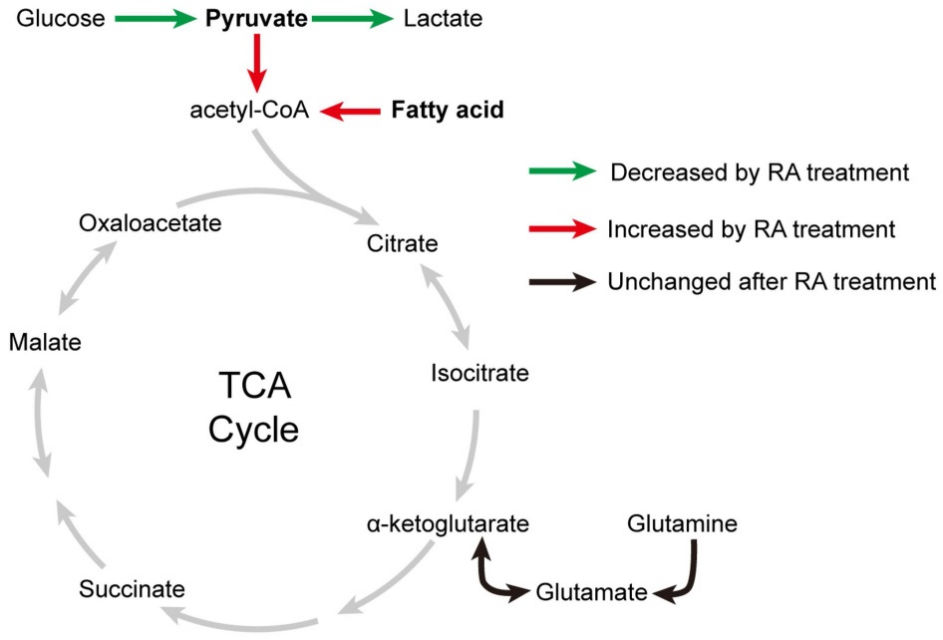
** Effects of RA on metabolic changes in cardiomyocytes.** After RA treatment on days 15-20, glycolysis in cardiomyocytes was decreased, (as indicated by the green arrows), whereas mitochondrial metabolism was increased, as evidenced by increased fatty acid and pyruvate usage (as indicated by the red arrows). These results supported the metabolic maturation of cardiomyocytes after RA treatment.

Taken together, our data indicate that RA promotes fatty acid and pyruvate metabolism in the mitochondria of hESC-CMs (Figure 9). However, due to the limited glycolysis and endogenous metabolite pyruvate in RA-treated hESC-CMs, RA mainly promotes the utilization of fatty acids in hESC-CMs in the absence of exogenous pyruvate.

## Discussion

Studies on embryo biology are valuable resources, providing direct evidence for the mechanisms of stem cell differentiation 17, 43, 44. As a ligand for the nuclear transcription factor RAR, RA exerts pleiotropic actions at different stages of heart development 14. However, RA is not essential for cardiomyocyte differentiation *in vitro*, because hESCs can differentiate into cardiomyocytes in chemically defined medium without RA or retinol 16, 23. Although the cardiomyocytes can be derived from hESCs, the differentiation efficiency and maturation of the derived cardiomyocytes still need to be improved. In this study, we identified two important stages for RA treatment to promote cardiomyocyte differentiation and maturation, respectively. We found that RA synthesis enzymes were primed in the primitive streak; however, *Stra6* was specifically expressed in the lateral mesoderm and was responsible for the transport of retinol into the cytoplasm, indicating that RA might be mainly synthesized in the lateral mesoderm after gastrulation. This result was consistent with the inhibitory function of RA treatment on primitive streak marker expression. Furthermore, we found that RA treatment on days 2-4 was important for lateral mesoderm development and downstream cardiomyocyte differentiation. Additionally, we observed RA treatment (days 2-4) promoted atrial-like cell specification and suppressed ventricular-like cell specification, consistent with a previous report 17.

During directed cardiomyocyte differentiation of human and mouse pluripotent stem cells, RA treatment after primitive streak formation was mainly used for atrial-like cardiomyocyte induction. However, the time interval for RA addition is not consistent at different induction conditions and in different species. In human PSC studies, Zhang Q *et al.* added RA (1 μM) on day 5-8 of differentiation to induce atrial-like cardiomyocyte in monolayer condition 18, whereas, Lee JH *et al*. added RA (500 nM) on day 3-5 using embryoid body-based condition 17. Besides, the inhibitors and cytokines used in these studies were not consistent because of different protocols, which may affect the time interval of RA treatment. In this study, we optimized the RA treatment time interval of day 2-4 in monolayer condition with chemical defined medium based on the development evidence. In addition to atrial-like cardiomyocyte specification, RA significantly enhanced cardiomyocyte differentiation. Thus, our study provided a novel insight to optimize the RA treatment time for maximal atrial-like cardiomyocyte induction at different induction conditions. In mouse ESC studies, RA treatment on day 5-15 promotes cardiomyocyte differentiation of ESC line D3 using embryoid body method 45, and addition of RA on day 5-15 promotes atrial-like cell differentiation in 129/Ola-derived ESC lines 46. Collectively, these human and mouse studies indicate RA concentration and treatment time interval should be optimized in different cell lines and different induction conditions to achieve the best effect.

Previous study has shown the functions of RA pathway during cardiac development and differentiation is conserved across species 17. Our results proved that RA promoted *KDR* expression during hESC differentiation. Consistently, RA induced *Kdr* expression during mouse ESC differentiation 47, indicating its conserved role in lateral mesoderm differentiation. RA pathway activation could induce atrial-like cardiomyocytes from pluripotent stem cells in mouse, monkey and human 18, 48, 49, which was also proved in current hESC study, consistent with its role for atrial chamber development in mouse embryo 15. Furthermore, we found RA promoted hESC-CM maturation, consistent with the structurally immature phenotype of ventricle cardiomyocytes in mouse RA-deficient embryos 15. These results support the conserved role of RA in cardiomyocyte maturation, but there may be some difference between mouse and human. *MHY7* is the dominant isoform in human adult heart, and the ratio of *MYH7*/*MYH6* was increased after RA treatment in our study. Whereas, *Myh6* is the dominant isoform in mouse adult heart 10. Whether mouse *Myh7*/*Myh6* is decreased by RA treatment needs to be investigated further.

Human ESC-CMs are powerful tools for heart disease modeling and drug screening 19, but their immature structures and metabolic properties have limited their application. In this study, although RA-treated hESC-CMs did not fully resemble adult-like cardiomyocytes, RA significantly promoted cardiomyocyte structural, electrophysiological and metabolic maturation. It is well known that RA exerts its effects through activation of RAR/RXR and PPAR/RXR heterodimers 50, and both of these receptors have been reported to promote fatty acid oxidation 51-53, supporting our results regarding the metabolic switch from glucose to fatty acid metabolism in RA-treated hESC-CMs. Moreover, the integration of RA and extracellular matrix supplementation may further accelerate hESC-CM maturation, because the extracellular matrix can promote the RAR expression and nuclear localization 54. This may be important for the full activation of RA signaling and needs to be further investigated.

Pyruvate dehydrogenase kinase 4 (*PDK4*) functions as an inhibitor of pyruvate dehydrogenase (PDH), which converts pyruvate into acetyl-coA and thereby increases the utilization of acetyl-coA from glycolysis in the TCA cycle. Overexpression of *Pdk4* in mouse hearts decreases glucose oxidation and increases fatty acid metabolism correspondingly 35. Therefore, its inhibitory effect on PDH activity reduces the use of pyruvate from glucose, and in turn resulted in the influx of acetyl-coA from fatty acid beta-oxidation into TCA cycle 43. In our study, we found that *PDK4* was significantly upregulated by RA treatment (Figure S9), which was consistent with the increased fatty acid oxidation in RA-treated hESC-CMs. Furthermore, although the glucose oxidation levels were low in both DMSO- and RA-treated groups overall, they were further reduced by RA treatment, consistent with PDK4 upregulation. When supplied with exogenous pyruvate, RA-treated hESC-CMs showed the elevated mitochondrial oxidative phosphorylation, inconsistent with *PDK4* upregulation. Thus, exogenous pyruvate metabolism may not depend on PDK4. In this study, we also found some splicing evidences, supporting the role of RA in cardiomyocyte metabolic maturation. *PKM* encodes pyruvate kinase and can generate *PKM1* and *PKM2* subtypes by alternative splicing of mutually exclusive exon 9 and 10, respectively. *PKM2* is expressed in the fetal heart, and is replaced by *PKM1* by postnatal day 14.5, indicating *PKM1* is related with cardiomyocyte maturation 38. The high activity PKM1 promotes the use of pyruvate in TCA cycle, while PKM2 facilitates pyruvate to convert to lactate 55, 56. The upregulation of PKM1 in RA-treated cardiomyocyte was consistent with the increased exogenous pyruvate metabolism in mitochondria. Thus, the pyruvate metabolism was regulated at multiple levels and needs further investigation. Collectively, the core function of RA on hESC-CM metabolism is promoting mitochondrial oxidative phosphorylation. This phenomenon was also found in RA-induced differentiated SH-SY5Y cells and adipocytes 57-59, indicating RA might play the conserved roles on metabolism in these cell lines.

Previous studies had demonstrated the RA receptor complex RAR/RXR could bind to RAREs and regulate target genes directly 14, 25, 60. Using ChIP-PCR, we found RAR could bind to the RAREs in *PGC1A* promoter and thus regulate metabolic gene directly. Whereas, ChIP-PCR did not identify the binding of RAR to *MYH6* and *MYH7* promoters (data not shown), indicating the effect of RA on *MYH7*/*MYH6* ratio might be indirect. Therefore, RA affects cardiomyocyte differentiation and maturation through both direct and indirect mechanisms.

In addition, the mechanism for RA-mediated splicing is still mysterious. It is reported that RA treatment can regulate RNA splicing through the ubiquitous nuclear protein Acinus in a dose- and time-dependent manner, and the splicing activity of Acinus is mediated by RA-activated RAR 61. Moreover, as a nuclear receptor coactivator, *PGC1A* affects splicing decisions 62. In our study, *PGC1A* was upregulated by RA treatment. Thus, RA treatment may affect splicing efficiency through these coregulators. However, the detailed mechanisms of RA in cardiomyocyte need to be further investigated.

In this study we found that RA promoted cardiomyocyte maturation in certain properties. RA-treated cardiomyocytes showed the improved maturation properties, including ultrastructure, cell area, oxidative metabolism, functional electrophysiology and calcium handling. However, these properties didn't reach the fully mature level of adult cardiomyocytes. As a single molecule, RA couldn't be expected to substantially induce hPSC-CM maturation like fully-developed cardiomyocytes isolated from adult heart, but it is an indispensable part of the orchestra. With the enrichment of the orchestra by different stimuli, including electromechanical stimulation, patterned biomaterials, developmental signal manipulation, metabolic stimulation and so on, fully mature cardiomyocytes might be obtained from hPSCs in the future 9, 10, 63, 64.

In conclusion, we found that RA was a critical factor for efficient lateral mesoderm differentiation in a confined time window, and subsequently promoted cardiomyocyte differentiation. More importantly, RA accelerated the structural, electrophysiological and metabolic maturation of hESC-CMs. Finally, we have provided a novel strategy to promote cardiomyocyte maturation through supplementation with RA.

## Supplementary Material

Supplementary figures and tables.

## Abbreviations

APD_50_: action potential duration at 50% repolarization
APD_90_: action potential duration at 90% repolarization
ChIP: chromatin immunoprecipitation
CM: cardiomyocyte
ECAR: extracellular acidification rate
ETO: etomoxir
FAO: fatty acid oxidation
FCCP: carbonyl cyanide 4-(trifluoromethoxy)phenylhydrazone
OCR: oxygen consumption rate
PGC1A: PPAR gamma coactivator 1-alpha
PSC: pluripotent stem cell
RA: retinoic acid
RAR: retinoic acid receptor
RARE: retinoic acid response element
Rtn/AA: rotenone and antimycin
TCA: tricarboxylic acid
