# Supplementary material for: Retinoic acid promotes metabolic maturation of human Embryonic Stem Cell-derived Cardiomyocytes
Source: Theranostics. 2020 Aug 1;10(21):9686–701. doi: 10.7150/thno.44146 (PMC7449904; doi:10.7150/thno.44146)
Supplement: Supplementary file 1 — Supplementary figures and tables. [file thnov10p9686s1.pdf]

SUPPLEMENTAL INFORMATION

Retinoic acid promotes metabolic maturation of human embryonic stem cell-derived cardiomyocytes

Shumei Miao[1, *], Dandan Zhao[1, *], Xiaoxiao Wang[2, 3, *], Xuan Ni[1, *], Xing Fang[1, *], Miao Yu[1], Lingqun Ye[1], Jingsi Yang[1], Hongchun Wu[1], Xinglong Han[1], Lina Qu[4], Lei Li[2, 3], Feng Lan[5, #], Zhenya Shen[1, #], Wei Lei[1, #], Zhen-Ao Zhao[6, #], Shijun Hu[1, #]

[1] Department of Cardiovascular Surgery of the First Affiliated Hospital & Institute for Cardiovascular Science, Collaborative Innovation Center of Hematology, State Key Laboratory of Radiation Medicine and Protection, Medical College, Soochow University, Suzhou 215000, China; [2] State Key Laboratory of Stem Cell and Reproductive Biology, Institute of Zoology, Chinese Academy of Sciences, Beijing 100101, China; [3] University of Chinese Academy of Sciences, Beijing 100049, China; [4] State Key Laboratory of Space Medicine Fundamentals and Application, China Astronaut Research and Training Center, Beijing 100094, China; [5] Fuwai Hospital, Chinese Academy of Medical Sciences and Peking Union Medical College, Beijing 100037, China;[6] Institute of Microcirculation & Department of Pathophysiology of Basic Medical College, Hebei North University, Zhangjiakou 075000, Hebei, China;

[*] These authors contributed equally to this work

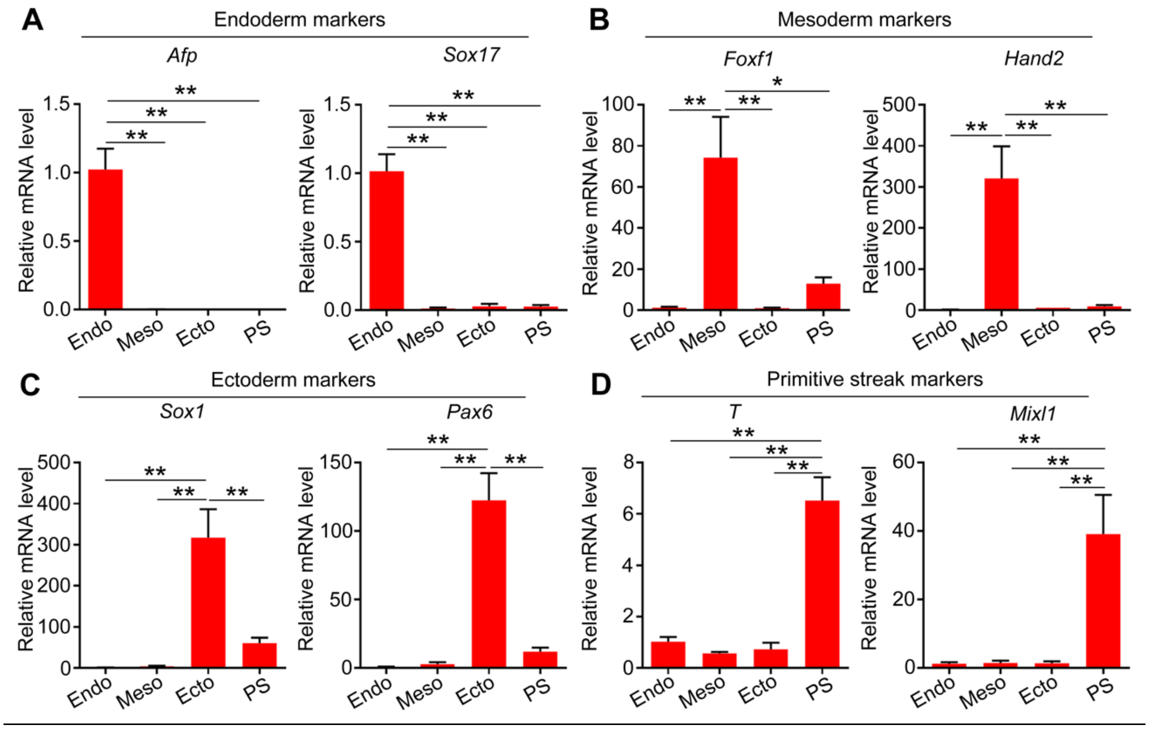

Figure S1. Germ layer marker expressions in dissected mouse embryo tissues. (**A**) Expression patterns of endoderm markers *Afp* and *Sox17*. (**B**) Expression patterns of mesoderm markers *Foxf1* and *Hand2*. (**C**) Expression patterns of ectoderm markers *Sox1* and *Pax6*. (**D**) Expression patterns of primitive streak markers *T* and *Mixl1*. Endo: endoderm; Meso: mesoderm; Ecto: ectoderm; PS: primitive streak. Student's *t*-test; *$p<0.05$; **$p<0.01$.

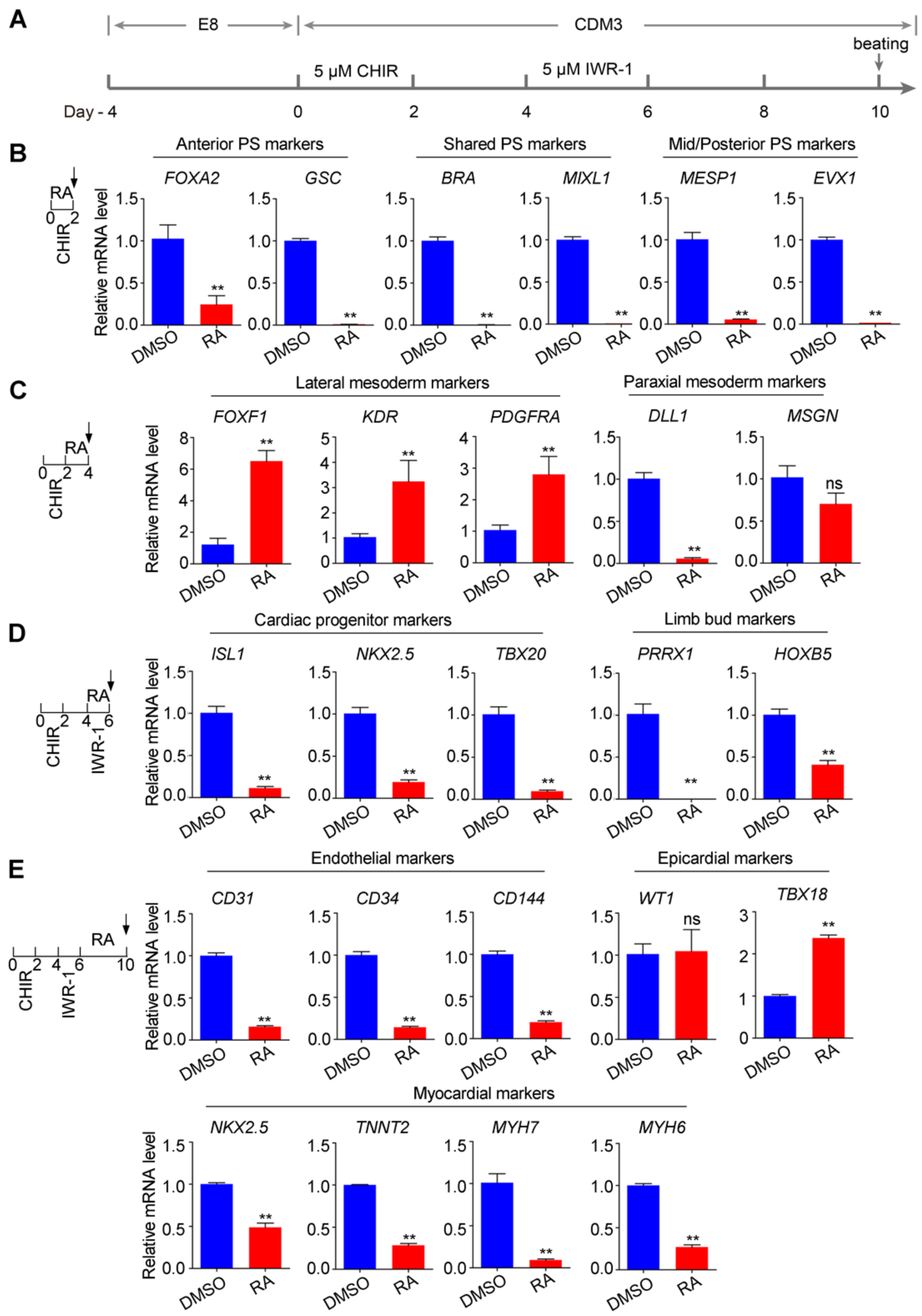

Figure S2. RA treatment during cardiomyocyte differentiation. (**A**) Schematic diagram of cardiomyocyte differentiation from hESCs. (**B**) Real-time PCR showed the expression levels of anterior PS, shared PS and mid/posterior PS markers in the DMSO- or RA-treated hESC-derived cells on day 2 of differentiation. PS: primitive streak. The downward arrow indicates the time point for qPCR detection. (**C**) Real-time PCR showed that RA promoted lateral mesoderm differentiation but not paraxial mesoderm differentiation. (**D**) Real-time PCR showed that cardiac progenitor markers and limb bud markers were significantly inhibited after RA treatment on days 4-6. (**E**) Real-time PCR showed that RA treatment on days 6-10 mainly inhibited the expression levels of endothelial and myocardial markers. Student's *t*-test; $^{**}p<0.01$, and ns, not significant.

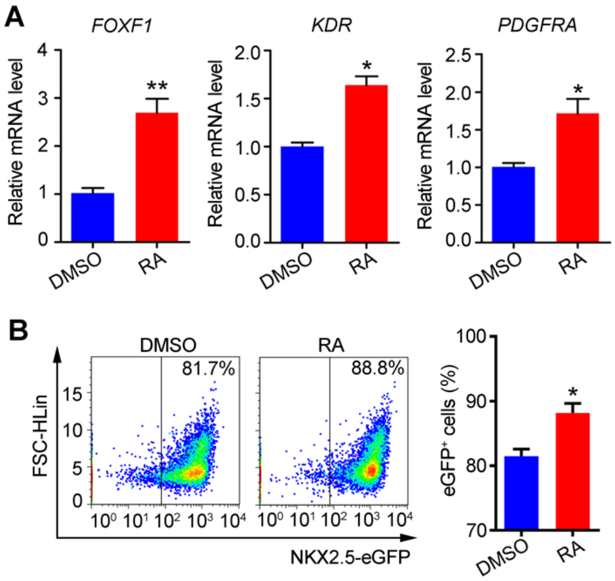

Figure S3. RA treatment on days 2-4 induces lateral mesoderm and cardiomyocyte differentiation in the NKX2.5$^{eGFP/W}$ hES3 line. (**A**) Real-time PCR showed that RA significantly increased lateral mesoderm markers on day 4 of differentiation. (**B**) Flow cytometry analysis of differentiated cardiomyocytes (day 10) showed that the proportion of NKX2.5-eGFP$^+$ cells was increased after RA treatment. Student's *t*-test; $^*p<0.05$; $^{**}p<0.01$.

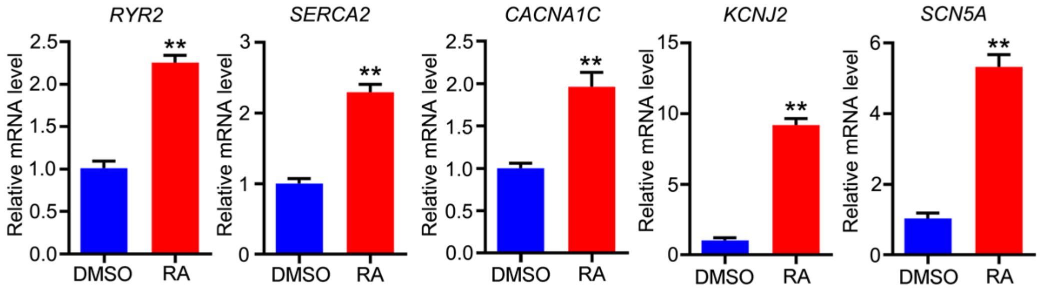

Figure S4. The effect of RA (days 15-20) on maturation marker expressions in cardiomyocytes derived from NKX2.5$^{eGFP/W}$ hES3 line on day 30. Student's *t*-test; **p<0.01.

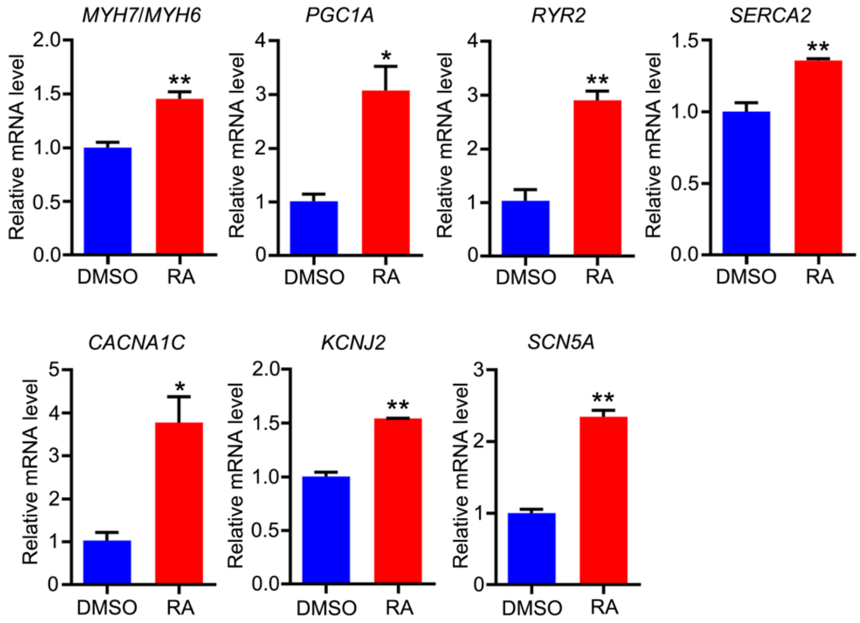

Figure S5. The effect of RA (days 15-20) on maturation marker expressions in cardiomyocytes derived from H1 hESC line on day 30. Student's *t*-test; $^{*}p<0.05$; $^{**}p<0.01$.

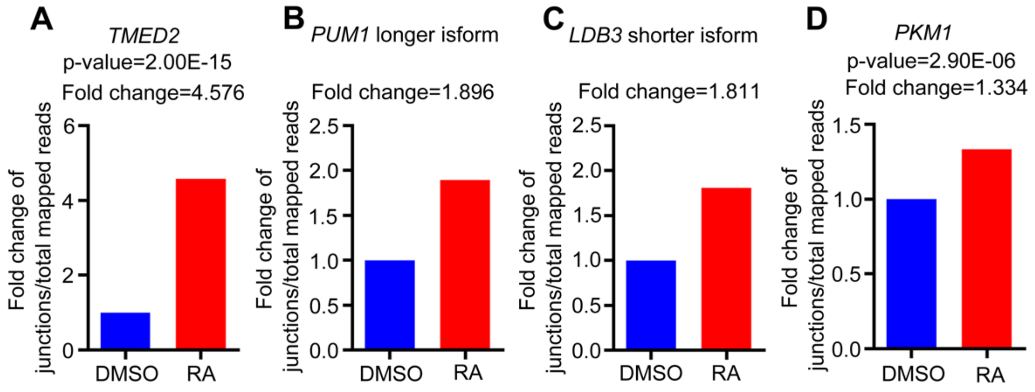

Figure S6. Quantification of RNA splicing.

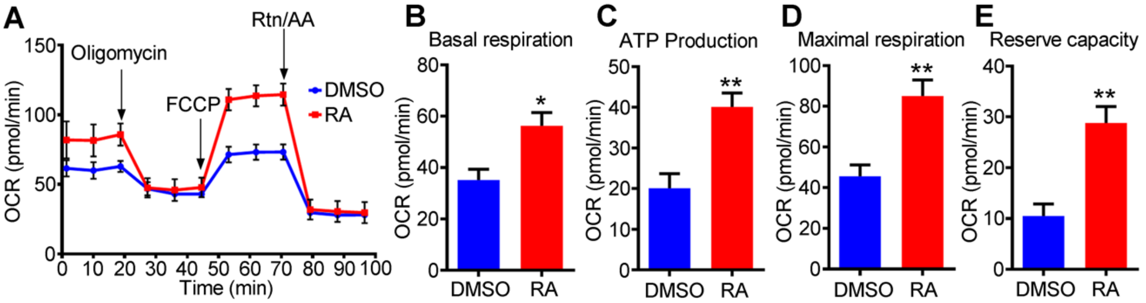

Figure S7. RA promotes the oxidative phosphorylation of cardiomyocytes derived from H1 hESC line. (A) Representative OCR traces of the DMSO- and RA-treated hESC-CMs derived from H1 ESCs in the presence of both glucose and pyruvate obtained using a Seahorse XF24 Extracellular Flux Analyzer. **(B-E)** Quantification of basal respiration (**E**), ATP production (**F**), maximal respiration (**G**) and reserve capacity (**H**). Student's *t*-test; $^{*}p<0.05$; $^{**}p<0.01$.

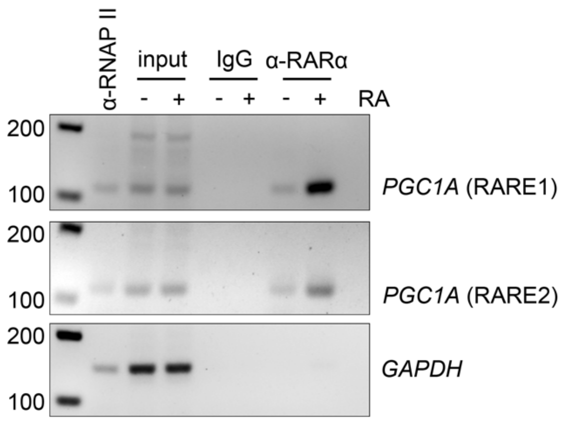

Figure S8. ChIP-PCR reveals the direct binding of RARα to RAREs in *PGC1A* promoter after RA treatment in hESC-CMs. The two RAREs in *PGC1A* promoter were named as RARE1 (-229 bp ~ -212 bp: 5'-AGGGTTATCTGGGGGCGA-3') and RARE2 (-70 bp ~ -53 bp: 5'-TGACTCTGAGATGCCCTC-3'), respectively.

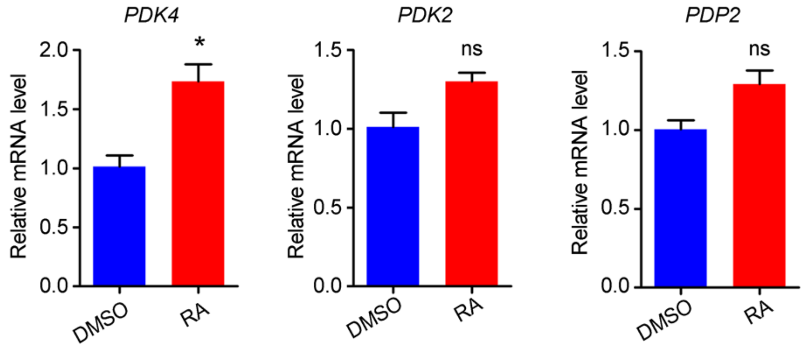

Figure S9. The effects of RA (days 15-20) on the expression of mitochondrial genes related to pyruvate metabolism. *PDK4*: pyruvate dehydrogenase kinase 4; *PDK2*: pyruvate dehydrogenase kinase 2; *PDP2*: pyruvate dehydrogenase phosphatase catalytic subunit 2. Student's *t*-test; $^{*}p<0.05$, and ns, not significant.

SUPPLEMENTAL TABLES

Table S1. Mouse primer sequences used for quantitative real-time PCR.

Genes	Forward	Reverse
Rbp1	AGGCATAGACGACCGCAAGT	ATCCACTGCGTCCAGCCAC
Aldh1a2	ATCGCTTCTCACATCGGCATAG	CCTCCGAGTTCCAGGGTCA
Crabp1	CATGCTGAGGAAGGTGGCC	TGAAGTTGATCTCCGTGGTGC
Cyp26a1	GGGTTTCGGGTTGCTCTG	CGGGATTAAATTCCTCCTTG
Rbp4	ACGAGTCCGTCTTCTGAGCAA	TGGAGAAAGGAGGCTACACCC
Stra6	TCGCCAAGCCATAGTCAGC	GCAGTAAAGGCACAAACACCAG
Afp	CAGCCAAAGTGGAGTGGAAAG	GGAAACTGGAAGGGTGGGAC
Sox17	CTTTATGGTGTGGGCCAAAG	TTCCAAGACTTGCCTAGCATC
Foxf1	GCATCCCTCGGTATCACTCAC	ATCCTCCGCCTGTTGTATGC
Hand2	TCCCCACCTCCCTCTCCA	CACACAGAGAATGACGGGGGT
Sox1	AGACTTCGAGCCGACAAGAG	AACTGTGCAAACAGGTGCAG
Pax6	TAACGGAGAAGACTCGGATGAAGC	CGGGCAAACACATCTGGATAATGG
T	CCAGAATGAGGAGATTACAGCC	TGGTCGTTTCTTTCTTTGGC
Mixl1	CGCTCCCTCAGTAACAACGC	GCTGCCACAGACTTCCAAATG
Gapdh	CCCCAATGTGTCCGTCGTG	TGCCTGCTTCACCACCTTCT

Table S2. Human primer sequences used for quantitative real-time PCR.

Genes	Forward	Reverse
STRA6	CCTACACGCTGCTGCACAA	GGAACATGCCTCAGCACAGA
FOXA2	AGGGCCAGAGTTCCACAAATC	GGGTATCCCTCCCTCCTTCTT
GSC	GAGGAGAAAGTGGAGGTCTGGTT	CTCTGATGAGGACCGCTTCTG
BRA	ACTACACACCCCTCACCCAT	GTACTGGCTGTCCACGATGT
MESP1	CCTGAAGGGCAGGCGATG	CCTTGTCACTTGGGCTCCTC
EVX1	AGTGACCAGATGCGTCGTTAC	TGGTTTCCGGCAGGTTTAG
FOXF1	AGCAGCCGTATCTGCACCAGAA	CTCCTTTCGGTCACACATGCTG
KDR	ATGTCAGAAAAGGAGATGCTCGC	TTTCCCACAGCAAAACACCAA
PDGFRA	AGAGGAACAGACACAGCTCG	TTCCACCAGGTCTGAAGAGTC
DLL1	ACTCCGCGTTCAGCAACCCCAT	TGGGTTTTCTGTTGCGAGGTCATCAGG
MSGN	CGGAATTACCTGCCACCTGT	GGTCTGTGAGTTCCCCGATG
ISL1	TACTGAGCGACTTCGCCTTG	GTGGAATTAGAGCCCGGTCC
NKX2.5	CTATCCGGGTTACGGCGG	TGAACCGCATTCAAGTCCCC
TBX20	TGCGGTGGGGAATAGAGG	GGGAGACAAAGACCCGAAAC
PRRX1	TGATGCTTTTGTGCGAGAAGA	AGGGAAGCGTTTTTATTGGCT
HOXB5	AACTCCTTCTCGGGGCGTTAT	CATCCCATTGTAATTGTAGCCGT
WT1	GCCTCACTCCTTCATCAAACA	GGCCGGAAAAGTGGACAGT
**TNNT2**	AGCGGAAAAGTGGGAAGAGG	CACAGCTCCTTGGCCTTCTC
MYH7	ACCTGTCCAAGTTCCGCAAG	TCATTCAAGCCCTTCGTGCC
MYH6	CAAGAGCCGTGACATTGGTG	AGGTTGGCAAGAGTGAGGTT
MYL7	GCCTTACACTGCTCTTTGGG	CAGATGAAGGGTGACGGGAG
IRX4	CAGGATAGCCGGAGACGC	TTAGGAGGTGGCTGAGACGG
SLN	AGTTAGATGAAGACCTACAGCAGC	GAAGGCGGCTATGTAAGATGAG
CD31	TTGAGACCAGCCTGATGAAACCCT	TCCGTTTCCTGGGTTCAAGCGATA
CD34	CAAGCCACCAGAGCTATTCC	TAGCCAGTGATGCCCAAGAC
CD144	CATCTTCCCAGGAGGAACAG	AGAGCTCCACTCACGCTCAG
TBX18	TCTGGCGACCATCACTACGG	GGGTGAGTGGCAGGAACG
PGC1A	GTCTAACTATGCAGACCTAGATTCA	CTGTCATCCTCAGCTAGGGAAC
ENO1	GGAGCAGGTTTACCACAA	CCTTCTTTATTCTCCAGGATGTT
ALDOA	GGCCTCCGTCTGGATTTC	GGGCATGGTGCTGGTAGTAG
LDHA	GACTTCTGAGGAAGAGGCCC	CATGCACAACCTCCACCTAGA
BPGM	AGGGAGAAAATGGCTTTGAATC	TCGTGGAAGTAGGGATGAGACT
PDK4	GAACCTGGCAAAAGAAGTGGC	AGGAGTTTTCGTTGCTGTCG
PDK2	ATGGCAGTCCTCCTCTCTGA	CACCCACCCTCTTCCTAACA
PDP2	GGTAGACGCTTATACTCCAGGT	CACATGGGGAACTGTTTAGGG
SERCA2	ACCTGGAACCTGTTCTTAGCTC	CATCACAGATGACAATTAGTGCC
RYR2	TCCGGAAACAGTATGAAGACCA	CACACAACGCTGGCAATTCA
CACNA1C	CATGCTCACGGTGTTCCA	TCCTACGGCATCATTGACC
KCNJ2	TGTCACGGATGAATGCCCAA	CTGCGCCAATGATGAAAGCA
SCN5A	TTCAGGGCTGAAGACCATCG	GCACTTGTGCCTTAGGTTGC
18S	GTAACCCGTTGAACCCCATT	CCATCCAATCGGTAGTAGCG
ND1 (mtDNA)	AACCTCAACCTAGGCCTCCT	GAGTTTGATGCTCACCCTGA
mtCO1 (mtDNA)	ACGTTGTAGCCCACTTCCAC	CATCGGGGTAGTCCGAGTAA
RNA18S5 (genomic DNA)	GCTGAGAAGACGGTCGAACT	CGCAGGTTCACCTACGGAAA

Table S3. Antibodies used in this study.

Antibody	Company	Catalog Number	Immunostaining	Flow cytometry	ChIP
Troponin T (TNNT2)	Thermo Scientific	MS-295-P1	1:200	1:200	
Sarcomeric Alpha Actinin (α-actinin)	Abcam	ab9465	1:200		
RARα	Abcam	ab41934			1: 1000
Alexa Fluor® 594 AffiniPure Donkey Anti-Mouse IgG (H+L)	Jackson ImmuoResearch	711-585-152	1:1000		
Alexa Fluor® 488 AffiniPure Donkey Anti-Mouse IgG (H+L)	Jackson ImmuoResearch	715-545-151	1:1000		
Alexa Fluor® 647 AffiniPure Donkey Anti-Mouse IgG (H+L)	Jackson ImmuoResearch	715-605-151	1:1000		

Table S4. Human primer sequences used for ChIP-PCR.

Target Region	Primer Sequence (5’→3’)	Product Length (bp)
Predicated RARE1	Forward: ATAACATGTATGCATGCC<br>Reverse: GTGAGTGTTCCCTCATCTCAT	119
Predicated RARE2	Forward: GTGCCCTATTGTGGAGTTC<br>Reverse：ATGAGATGAGGGAACACTCAC	119
